# Increased FDG avidity in lymphoid tissue associated with response to combined immune checkpoint blockade

**DOI:** 10.1186/s40425-016-0162-9

**Published:** 2016-09-20

**Authors:** Katy K. Tsai, Miguel H. Pampaloni, Charity Hope, Alain P. Algazi, Britt-Marie Ljung, Laura Pincus, Adil I. Daud

**Affiliations:** 1 Helen Diller Family Comprehensive Cancer Center, University of California, San Francisco, 1600 Divisadero Street, Box 1770, San Francisco, CA 94115 USA; 2 Department of Radiology and Biomedical Imaging, University of California, San Francisco, 505 Parnassus Avenue, San Francisco, CA 94117 USA; 3 Department of Dermatology, University of California, San Francisco, 1701 Divisadero Street, San Francisco, CA 94115 USA; 4 Department of Pathology, University of California, San Francisco, 1600 Divisadero Street, San Francisco, CA 94115 USA

**Keywords:** Immunotherapy, Biomarker, PD-1, CTLA-4, PET/CT

## Abstract

**Background:**

Antibodies against programmed death 1 (PD-1) receptor and cytotoxic T-lymphocyte-associated antigen 4 (CTLA-4) have transformed the systemic treatment of melanoma and many other cancers. Understanding the spectrum of benign findings and atypical response patterns seen in immune checkpoint blockade is important for accurately assessing treatment response as these immunotherapies become more widely used.

**Case presentation:**

We report a 63-year-old man with metastatic melanoma successfully treated with combination CTLA-4 and PD-1 blockade (ipilimumab and nivolumab), after non-response to pembrolizumab monotherapy. The initial impression of disease progression, based on cutaneous and PET/CT findings of increased fluoro-2-deoxy-D-glucose (FDG) uptake in benign lymphoid tissue, proved to be erroneous after assiduous review of radiographic imaging and correlative pathology.

**Conclusions:**

These findings indicate that increased FDG uptake in benign lymphoid tissue seen on PET/CT may be a surrogate marker of immune activation and treatment response. Prospective studies will be invaluable in validating immune-related radiographic findings as a prognostic biomarker of response in cancer patients being treated with immune checkpoint blockade.

## Background

Melanoma is an aggressive cutaneous neoplasm. Surgical resection for early stage melanoma is often curative, but metastatic disease carries a poor prognosis, highlighting the importance of accurate staging. Whole-body positron emission tomography (PET) with 2-[fluorine-18]-fluoro-2-deoxy-D-glucose (FDG) combined with computed tomography (CT) scans demonstrate improved sensitivity and specificity for melanoma detection, and their utility in melanoma staging and monitoring is well established [1–5]. As systemic treatments for melanoma have advanced, there has been increasing interest in using FDG avidity not only for staging and monitoring, but as a predictive biomarker to assess prognosis and effectiveness of therapy [6].

We present a case of increased FDG uptake in lymphoid tissue as a surrogate marker of immune activation in a patient with metastatic melanoma treated with combination ipilimumab and nivolumab, following non-response to pembrolizumab monotherapy. Our discussion includes details of radiographic imaging and pathologic analyses.

## Case presentation

A 63-year-old man came to the University of California, San Francisco in May 2015 seeking care for his melanoma. Review of outside medical records revealed that in August 2014, a biopsy of a left scalp lesion showed a non-ulcerated, 1.3 mm thick melanoma. He subsequently underwent wide local excision (WLE) and sentinel lymph node biopsy (SLNB). The SLNB was positive for a 4 mm focus of BRAF wild-type melanoma in the subcapsular region of a left cervical lymph node. A PET/CT scan did not show any metastatic lesions.

During his consultation at UCSF, the initial diagnosis of stage IIIA melanoma was confirmed. Unfortunately, in the interim he had developed new hyperpigmented papules on his left chest. One was biopsied on 5/19/15, and pathology demonstrated features of epidermotropic metastatic melanoma. He was upstaged to stage IV melanoma, and on 7/1/15 was started on a clinical trial of intralesional IL-12. He had progressive lesions after 12 weeks on therapy, and on 9/15/15 was transitioned to standard of care pembrolizumab. He received two doses (10/5/15, 10/26/15) which were well tolerated, but had rapidly increasing size and number of hyperpigmented papules and plaques across his chest, bilateral arms and back. On 11/16/15 he received his first dose of combination ipilimumab/nivolumab. A PET/CT scan on 11/20/15 did not show any lesions concerning for metastasis. He went on to receive four induction doses of ipilimumab/nivolumab; a PET/CT scan was repeated on 2/11/16 and he was transitioned to nivolumab monotherapy. At this time, the patient remains on nivolumab and is tolerating treatment well.

## Methods

### Radiologic technique

Patient’s fasting time was at least six hours. Following intravenous administration of F18-FDG, CT was acquired from vertex to toes with intravenous contrast (Omnipaque 350), followed by an emission PET scan. A rotating 3D maximum intensity projection image, as well as axial, coronal, and sagittal PET images were interpreted.

### Diagnostic dermatopathology

Punch biopsies of cutaneous chest lesions were obtained prior to and during immunotherapy. Immunohistochemical staining was performed with antibodies against Melan-A (clone A103; dilution 1:200) (Dako, Carpinteria, CA).

### Diagnostic fine needle aspiration

Inguinal lymph nodes were visualized with ultrasound imaging. Targets were oriented in a position that allowed sampling throughout the hypoechoic cortex. On-site smear evaluation showed adequate material.

## Results

### Clinical findings

**Fig. 1:**
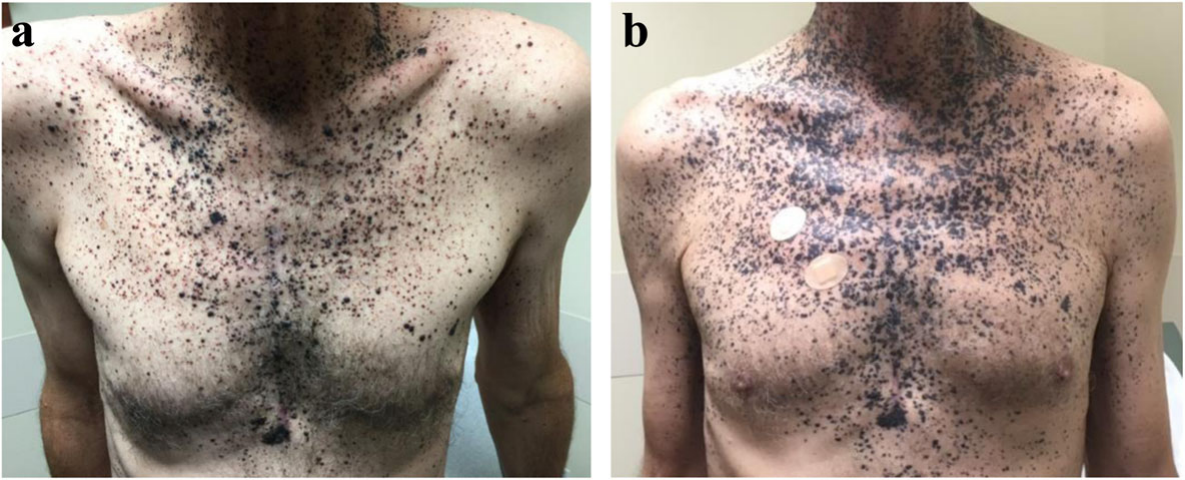
Clinical photographs prior to and during treatment with immune checkpoint inhibition. Panel **a** shows a photograph of the chest from 10/5/15 prior to initiation of pembrolizumab, with numerous hyperpigmented papules and plaques scattered across the chest. Panel **b** shows a photograph of the chest from 2/23/16, after completing ipilimumab/nivolumab induction. There is increased confluence of existing lesions, as well as new hyperpigmented papules on the bilateral upper arms and the neck

The patient had widespread cutaneous metastases that were readily clinically observed though not detected on PET/CT. His photograph on 10/5/15, prior to pembrolizumab, shows hyperpigmented papules scattered on the chest and neck (Fig. 1a). While receiving pembrolizumab, he developed increasing confluence of existing chest papules, and numerous new hyperpigmented papules on the neck, abdomen and bilateral upper arms. His treatment was changed to ipilimumab/nivolumab on 11/16/15 due to clinical concern for progressive disease. Upon completion of ipilimumab/nivolumab induction, clinical photographs were repeated on 2/23/16 (Fig. 1b). Although his appearance continued to be concerning for progression, there were subtle improvements (absence of new lesions, flattening papules) towards the end of the induction period.

### Radiologic findings

**Fig. 2:**
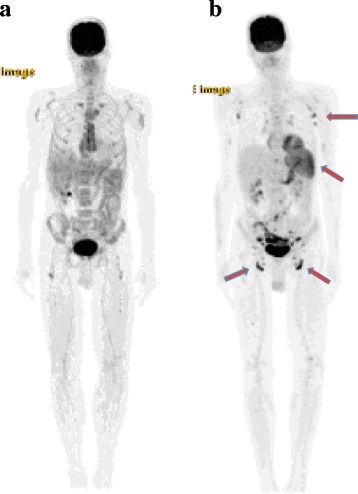
Maximum intensity projection images of two whole-body 18 F-FDG PET/CT studies. Panel **a** shows the PETCT from 11/20/15, early in the course of immunotherapy. Panel **b** shows the PET/CT from 2/11/16, after completion of induction ipilimumab/nivolumab. *Arrows* indicate the increased metabolic glucose consumption in bilateral inguinal, iliac, axillary lymph nodes as well as the spleen, highly suggestive of immune activation

The PET/CT on 11/20/15, four days after the first dose of ipilimumab/nivolumab, showed no remarkable areas of FDG avidity (Fig. 2a). The PET/CT on 2/11/16 showed hypermetabolic lymph nodes throughout multiple nodal stations: in bilateral inguinal, iliac, and axillary lymph nodes as well as in the spleen (Fig. 2b). The inguinal lymph nodes were the largest, measuring 8 mm in short axis, and with increased FDG avidity (12 SUV_max_). There was initial concern for progressive disease, however the bilateral pattern of uptake, preservation of fatty hilum, and concurrent splenic hypermetabolism (8.4 SUV _max_) combined with clinically slowed growth and flattening of cutaneous lesions were suggestive of FDG avidity as a marker of immune activation rather than progressive disease.

### Pathologic findings

**Fig. 3:**
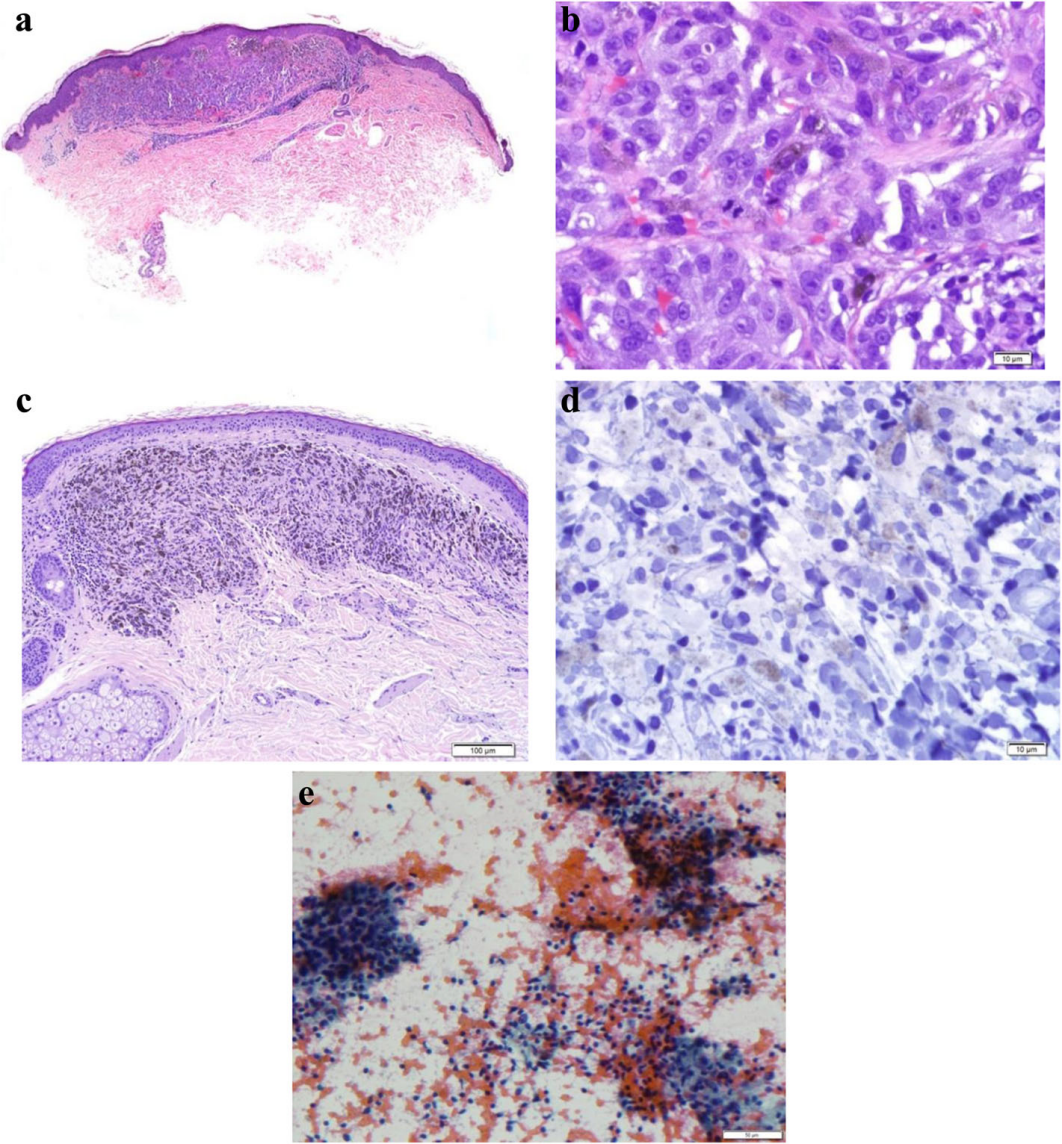
Punch biopsies of cutaneous lesions of the chest before (Panels **a** and **b**) and during (Panels **c** and **d**) systemic immunotherapy. Panel **a** shows a narrow-based compound melanocytic proliferation at low power (4×) with a few nests of melanocytes within the lower epidermis and a few melanocytes above the basal layer of the epidermis. In addition, there are nests of melanocytes within the dermis and mitotic figures are present among them. These features are consistent with metastatic melanoma. Panel **b** shows a high-power view (60×) of the melanocytes in the dermis, highlighting scattered dermal mitoses. Panel **c** shows a biopsy on treatment at low power (10×) with an area of complete regression of metastatic melanoma with dermal fibrosis and numerous melanophages. A high-power view (60×) of a Melan-A immunoperoxidase stain, shown in Panel **d**, does not show any labeling, confirming that there is no evidence of residual melanoma. Panel **e** shows the cytology smear from the inguinal node fine needle aspiration at 200×, with predominantly small unremarkable lymphocytes

The skin biopsy on 5/19/15, prior to pembrolizumab therapy, revealed a narrow diameter compound proliferation of melanocytes (Fig. 3a). There were nests of melanocytes within the lower epidermis and scattered single melanocytes in irregular pagetoid array above the basal layer of the epidermis. There were similar melanocytes in the dermis, and mitotic figures were evident among the dermal melanocytes (Fig. 3b). The melanocytes in the dermis generally did not diminish in size with descent. These findings were consistent with epidermotrophic metastatic melanoma.

A punch biopsy was obtained of cutaneous lesions on the right chest on 2/18/16, which showed melanophages and fibrosis. A Melan-A immunostain confirmed that no melanocytes were present in the dermis. These features were consistent with complete regression of melanoma (Fig. 3c, d).

Fine needle aspiration of bilateral inguinal lymph nodes was done on 2/17/16. Pathology yielded unremarkable small lymphocytes, without evidence of malignancy [7, 8] (Fig. 3e).

## Conclusions

Monoclonal antibodies against PD-1 and CTLA-4 are approved for advanced melanoma, non-small cell lung cancer, and renal cell carcinoma, and are being investigated in numerous other cancers. Use of immunotherapies is poised to increase in the coming years, thus highlighting the need for clinicians to gain expertise in immunotherapeutic response patterns.

Historically, the Response Evaluation Criteria in Solid Tumors (RECIST) have been the most widely accepted guidelines for defining solid tumor progression [9, 10]. However, atypical response patterns have been demonstrated in clinical trials of ipilimumab [11] and pembrolizumab [12], and have led to the development of immune-related response criteria (irRC) [11]. Described patterns of atypical response, or pseudoprogression, have included initial increases in the size of target lesions followed by decreases without the appearance of new lesions, and regression of target lesions but with appearance of new lesions. Benign lymphadenopathy has also been described as a radiographic finding in melanoma patients treated with anti-CTLA-4 therapy, the most common pattern reported to be sarcoid-like and in a bilateral hilar and mediastinal distribution. These findings were seen in a series of eight patients on anti-CTLA-4 therapy; there was no reported detail on FDG avidity. Interestingly, in the same series, radiologic evidence of immune-related effects seemed to correlate with response to treatment [13]. More recent efforts to identify an imaging biomarker have reported an elevated spleen-to-liver SUV ratio to be associated with increased overall survival in metastatic melanoma patients treated with anti-CTLA-4 or anti-PD-1 therapy [14].

Our case is the first report of FDG-avid diffuse lymphadenopathy occurring with combined CTLA-4 and PD-1 blockade, with correlative pathology confirming benign lymphadenopathy and regression of cutaneous metastases. The initial clinical impression of disease progression proved to be erroneous after assiduous review of radiographic imaging and biopsy results. This highlights the importance of multi-disciplinary evaluation of these cases, and the need for more prospective work to evaluate FDG avidity of benign lymphoid tissue in a larger case series as a surrogate for clinical benefit [15]. Heightened awareness of the full spectrum of benign findings and atypical response patterns seen with immunotherapies will improve our care of cancer patients being treated with these increasingly widespread agents, and encourage further research and development of novel imaging biomarkers.

## Abbreviations

CT: Computed tomography
CTLA-4: Cytotoxic T-lymphocyte-associated antigen 4
FDG: Fluoro-2-deoxy-D-glucose
irRC: Immune related response criteria
PD-1: Programmed death receptor-1
PET: Positron emission tomography
RECIST: Response evaluation criteria in solid tumors
SLNB: Sentinel lymph node biopsy
SUV: Standardized uptake value
WLE: Wide local excision
